# H-plane horn antenna with enhanced directivity using conformal transformation optics

**DOI:** 10.1038/s41598-021-93812-6

**Published:** 2021-07-12

**Authors:** Hossein Eskandari, Juan Luis Albadalejo-Lijarcio, Oskar Zetterstrom, Tomáš Tyc, Oscar Quevedo-Teruel

**Affiliations:** 1 Department of Electrical Engineering, Ferdowsi University of Mashhad, Mashhad, Iran; 2 Division of Electromagnetic Engineering, KTH Royal Institute of Technology, Stockholm, Sweden; 3 Department of Theoretical Physics and Astrophysics, Faculty of Science, Masaryk University, Kotlár̆ská 2, 61137 Brno, Czechia

**Keywords:** Electrical and electronic engineering, Transformation optics, Materials for devices

## Abstract

Conformal transformation optics is employed to enhance an H-plane horn’s directivity by designing a graded-index all-dielectric lens. The transformation is applied so that the phase error at the aperture is gradually eliminated inside the lens, leading to a low-profile high-gain lens antenna. The physical space shape is modified such that singular index values are avoided, and the optical path inside the lens is rescaled to eliminate superluminal regions. A prototype of the lens is fabricated using three-dimensional printing. The measurement results show that the realized gain of an H-plane horn antenna can be improved by 1.5–2.4 dB compared to a reference H-plane horn.

## Introduction

Ever since transformation optics (TO) was proposed by Pendry^1^ and Leonhardt^2^ in 2006, it has been used in a plethora of applications, like carpet cloaking^3–5^, polarization splitting and transforming^6–10^, waveguide coupling^11–17^, lens compression^18–21^, lens flattening^22–24^, and directivity enhancement^25–31^. TO provides a systematic approach to finding a material that realizes a particular functionality, which is essentially an inverse problem. This method establishes a correspondence between the electromagnetic fields and media of two spaces: virtual and physical. The two spaces are geometrically related to each other by a mapping. Based on the form-invariance of Maxwell’s equations, TO implies that a calculated transformation medium can mimic space deformation.

The material derived by the general types of transformation is quite complex and may be difficult to realize. The transformation medium is, in many cases, nonhomogeneous, anisotropic, and possesses extreme permittivity and permeability values. Such media can be realized with the use of resonant metamaterials, which inevitably limit the bandwidth of operation. In contrast, all-dielectric graded-index materials (GRIN) are advantageous in terms of realization and can operate over a wide bandwidth. GRIN devices can perform a variety of functions, such as waveguide mode conversion^32,33^, waveguide splitting^34^, and cloaking within a waveguide^35^. Also, they can act as an acoustic retroreflector^36^ and can support Fano resonances inside a waveguide^37^. To enhance the practicality of TO designs and achieve a GRIN media, conformal TO (CTO) and quasi-conformal TO (QCTO) are employed. The transformation used in the case of CTO is analytical. Such property removes the anisotropy from the transformation medium and makes the material all-dielectric for 2D cases where the electric field polarization is out-of-plane.

Horn antennas are among the simplest and most common types of antennas. A metallic tapering is introduced to ensure proper impedance matching while providing high directivity. These antennas are used as reflector feeds, phased array elements, calibration antennas for measurements, etc. H-plane horns are low-profile since the tapering is applied only to the H-plane. Such a slim antenna can be used as an array element. There are methods to improve the radiation characteristics of a horn. For example, using corrugations, which is common in conical antennas, can help attain lower sidelobe, backlobe, and cross-polarization levels as well as a better radiation pattern symmetry. All these features are required when using a horn as a reflector feed, especially in satellite applications^38^. Also, using profiled boundaries can make the horn shorter and provide better phase center stability^39^. The directivity of the horn can be enhanced by using longitudinal strips and dielectric fillings (hard horn) or stepped tapers (multi-mode horn)^40–42^.

An effective way to enhance the directivity is to place a homogeneous dielectric lens at the antenna aperture. While such lenses reduce the phase error at the aperture, they make the antenna bulkier and introduce intrinsic reflections due to the impedance mismatch^39^. Using anti-reflection (AR) layers can reduce the reflections at the expense of limiting the bandwidth and increasing the fabrication complexity. However, in the case of GRIN lenses, the functionality depends more on the refractive index distribution of the lens rather than its shape. Using GRIN lenses, the mismatch can be mitigated by engineering the impedance, and also, the profile of the lens antenna can be smaller compared to conventional dielectric lenses. Using TO, one can design a graded-index lens that is conformal to the horn’s body. Hence, the lens is embedded in the horn, leading to a compact horn lens antenna.

Several works in the literature employ CTO and QCTO to enhance the directivity of antennas^26–31^. In these works, the transformation is applied to design a lens that mitigates the phase error at the device aperture. Using this approach, the rays arrive at the aperture in-phase and at perpendicular angles. However, there are three common shortcomings for applying CTO and QCTO to the case of lens antennas. First, the device’s refractive index lacks matching to free-space at the aperture since the cylindrical to plane wave conversion is realized in the TO design by mapping a curved phase front line to a straight one, which inherently leads to a non-unity refractive index. If the source is not embedded in the transformation regions and a lens cap is designed, reflections occur on both sides of the lens^29^. The reflection can be mitigated by using an AR coating. However, apart from making the structure frequency-dependent, the fabrication process is complex since the non-uniform refractive index at the aperture requires an AR coating with a non-uniform index and a non-uniform thickness^43^. Second, since the virtual space is stretched in some regions to fit the physical space shape, the refractive index falls below unity, meaning that some superluminal regions are created. Third, the refractive index can obtain singular values at the corners^26–29^. The superluminal regions and the extreme refractive index values at the corners are often neglected and substituted with an index of one^27–30^. However, this deteriorates the aperture efficiency since the new unity-index area does not contribute to the phase error compensation.

In this work, we address all of the above shortcomings to enhance the directivity of an H-plane horn antenna while keeping its refractive index at moderate, above-unity values. This is achieved by applying CTO in such a way that the refractive index at the aperture matches that of the environment, keeping reflections to a minimum while preventing extreme refractive index values at the corners. Besides, any superluminal regions are remedied by rescaling the optical path^44^. To prove the functionality of our design method, we fabricated a prototype of the lens using a 3D printer and evaluated its performance in the ${\mathrm{{K}}_{\mathrm{{u}}}}$ band. It is seen that the realized gain can be increased by 1.5-2.4 dB.

## Design method

The 2D virtual and physical spaces are described by Cartesian coordinates (*u*,*v*) and (*x*,*y*), respectively. If we define the complex variables $W=u+\mathrm{i}v$ and $Z=x+\mathrm{i}y$, the analytical function *Z*(*W*) describes the conformal transformation from virtual to physical space. If the refractive index of the virtual space is *n*(*u*, *v*), then the refractive index of the physical space *n*(*x*, *y*) is derived using the well-known TO formula^2^:

1 $$\begin{aligned} n(x,y) = \sqrt{\varepsilon (x,y)} = n(u,v)\left| {\frac{{\mathrm{{d}}W(Z)}}{{\mathrm{{d}}Z}}} \right| = n(u,v)\sqrt{u_x^2 + u_y^2}. \end{aligned}$$

**Figure 1:**
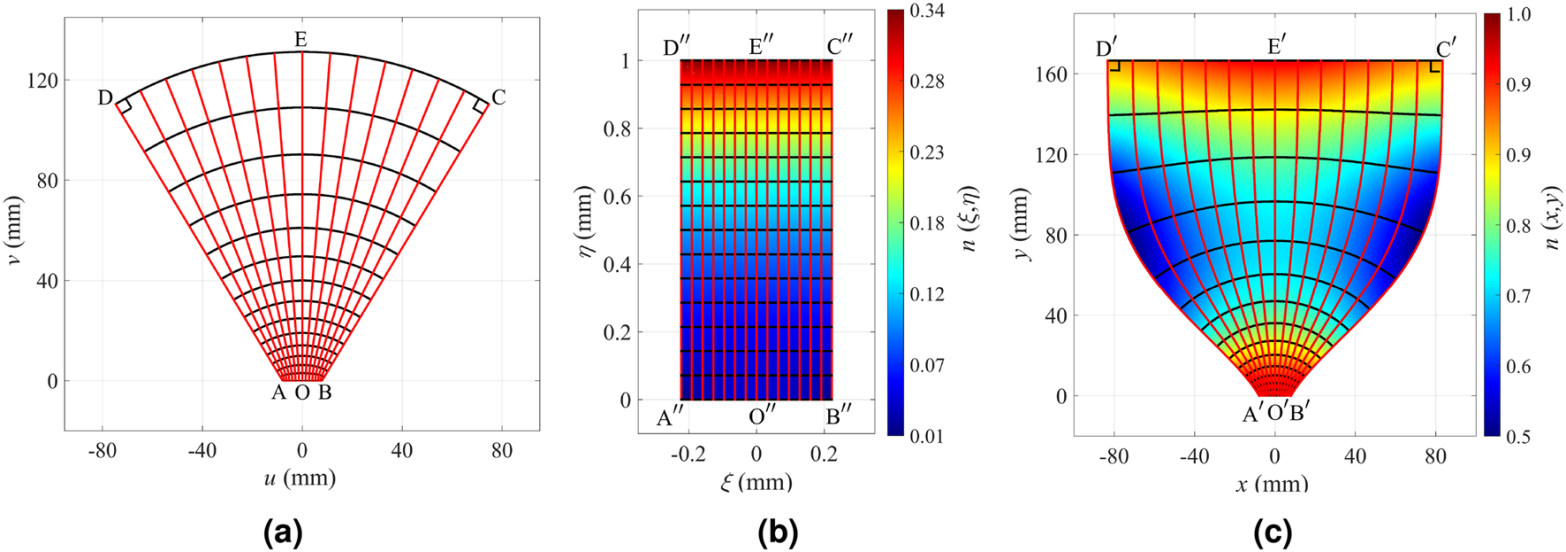
The schematics of (**a**) the virtual space, (**b**) the intermediate space and its refractive index, and (**c**) the physical space and its refractive index. The constant $\eta$ and $\xi$ coordinate lines in the intermediate space and their corresponding mapped images in the virtual and physical spaces are depicted by black and red lines, respectively.

Figure 1 illustrates the mapping procedure for the design of a directive CTO horn antenna. The coordinates of virtual and physical spaces are assigned as (*u*, *v*) and (*x*, *y*). In order to map the virtual space to the physical space, a canonical intermediate mapping domain is chosen. The most common canonical shapes are a rectangle and a unity circle; we choose a rectangle as the canonical intermediate region, and denote its coordinates as $(\xi ,\eta )$. Based on the Riemann theory of conformal mapping, all quadrilaterals are conformally equivalent if and only if they share the same conformal module^45^. The conformal module of a rectangle is its aspect ratio.

The fully-employed CTO does not induce reflections in principle as it is derived directly by applying a coordinate transformation to the 2D Helmholtz equation. However, if a ‘finite embedded’ transformation is employed^46^ or the physical space domain is curtailed for practical purposes, an intrinsic boundary reflection is created^47^. This reflection is inherently induced, although it may be negligible depending on the design requirements. Part of the reason is that the transformation continuity required for the reflectionless property at the input and output boundaries of the device can not be fully satisfied due to the uniqueness theorem^48 ^(Theorem 10.39). That is, if the transformation continuity is satisfied on the outer boundary of a transformed region, then the transformation should be a unity transformation everywhere, which is not the case with CTO designs. As we demonstrate in our design method, the boundary reflections can be reduced by carefully considering some factors.

Note that although the CTO is essentially defined for 2D cases, it is applicable to quasi-2D cases such as H-plane horn antennas. For the case of H-plane horn antennas, the guided mode excited by the feeding waveguide is the $\mathrm{{T}}{\mathrm{{E}}_{10}}$ mode. This mode does not have vertical variations, i.e., it is uniform in the *z*-direction, complying with the 2D assumptions of the CTO. This is the main mode excited in the horn. Also, the wavefronts of the guided mode inside an empty horn’s body closely resemble cylindrical contours^49^. As a result, in our design method, the virtual space depicted in Fig. 1a is the body of a horn that is filled with a vacuum. The horn’s throat matches the WR62 waveguide with a length of 15.8 mm, and the flare angle equals 31 $^\circ$. The physical space depicted in Fig. 1c is a region similar to the virtual space with two significant differences. First, the *cd* edge is flattened into ${c^\prime }{d^\prime }$ that eventually leads to the directivity enhancement. Second, the side boundaries are curves rather than straight lines. We have carefully defined ${b^\prime }{c^\prime }$ and ${d^\prime }{a^\prime }$ curves. The curve slope matches the flare angle near the throat and has zero first and second derivatives, $\mathrm{d}x/\mathrm{d}y$ and $\mathrm{d}^2x/\mathrm{d}y^2$, at the aperture. The zero first derivative ensures that the aperture corner angles are 90 $^\circ$ in all three spaces, which helps us avoid singular refractive index values near the corners. The zero second derivative increases the uniformity of the refractive index at the aperture. To avoid extreme refractive indices near the corners, the angles at the $\mathrm{{A}}$, $\mathrm{{B}}$, $\mathrm{{C}}$, and $\mathrm{{D}}$ corners must equal the ones of $\mathrm{{A'}}$, $\mathrm{{B'}}$, $\mathrm{{C'}}$, and $\mathrm{{D'}}$, respectively. The throat’s width of the horn antenna in the physical space is similar to the virtual space’s one ($\mathrm{{AB}}$=$\mathrm{{A'B'}}$) to get the refractive index close to one. In the physical space, the aperture has a length of 167 mm, which corresponds to 8.35 free-space wavelengths at the frequency of 15 GHz. The lens antenna length $\mathrm{{O'E'}}$ equals 167 mm.

By solving a boundary-value problem, the physical space’s conformal module is calculated, and it equals 0.44^44^. Hence, the intermediate rectangle aspect ratio is known, and we choose a unit length for its longer side. The *bc* side’s length is tuned to $bc=129$ mm to make the conformal module of the virtual space equal to 0.44. The conformal module equality in all three spaces guarantees the existence of the conformal map.

Knowing the dimensions of all three spaces, the mapping from the virtual space to the physical space is conducted by solving Laplace’s equation in the virtual and physical spaces separately for the intermediate space $\xi$ and $\eta$ coordinates and combining the results of the mapping. The intermediate region’s refractive index is calculated by establishing the conformal mapping between the virtual space and the intermediate space shown in Fig. 1b. Then, the physical space’s final refractive index is derived by calculating the conformal mapping between the intermediate space and the physical space. The refractive index formula follows:

2 $$\begin{aligned} n(x,y) = \frac{{\sqrt{\eta _x^2 + \eta _y^2} }}{{\sqrt{\eta _u^2 + \eta _v^2} }}\,, \end{aligned}$$

where the denominator is the refractive index of the intermediate space. The refractive index of the physical space is depicted in Fig. 1c. It is seen that the refractive index is below unity in some areas. Having established the correspondence of all points in all three spaces, the constant $\eta$ and $\xi$ coordinate lines in the intermediate space and their corresponding mapped lines in the virtual and physical spaces are illustrated in Fig. 1 by black and red lines, respectively.

The only remaining issue is the superluminal area, which can be mitigated by the following optical path rescaling method^44^. Consider one of the rays in Fig. 1c (that are shown in red). We can define an optical path associated with any point A lying on that ray as an integral

3 $$S(A) = \int_{{{\text{A}}_{0} }}^{{\text{A}}} n {\text{d}}l.$$

Here, the integral is taken along the ray from point A$_0$ to point A. A$_0$ is a point on the ray for which the optical path is set to zero. In our case, A$_0$ is the intersection of the ray with the line $a'b'$ where we define the optical path to be zero. The optical path defined in this way is proportional to the phase of the wave associated with the rays; the proportionality factor is $1/k=c/\omega$. Since each point of the physical space lies on exactly one ray (according to our design of the rays in Fig. 1c), we can define the optical path *S*(*x*, *y*) in the whole physical space. The refractive index then satisfies $n(x,y)=|\nabla S(x,y)|$, and the direction of $\nabla S(x,y)$ is tangent to the ray passing through the given point.

Now, suppose we define an increasing function $S'(S)$ that we call the “rescaled optical path”. If we imagine that $S'$ rather than *S* describes the optical path, then the corresponding refractive index will be

4 $$\begin{aligned} n'(x,y) = |\nabla S'|=|\nabla S|\,\frac{\mathrm{d}S'}{\mathrm{d}S}=n\,\frac{\mathrm{d}S'}{\mathrm{d}S}. \end{aligned}$$

**Figure 2:**
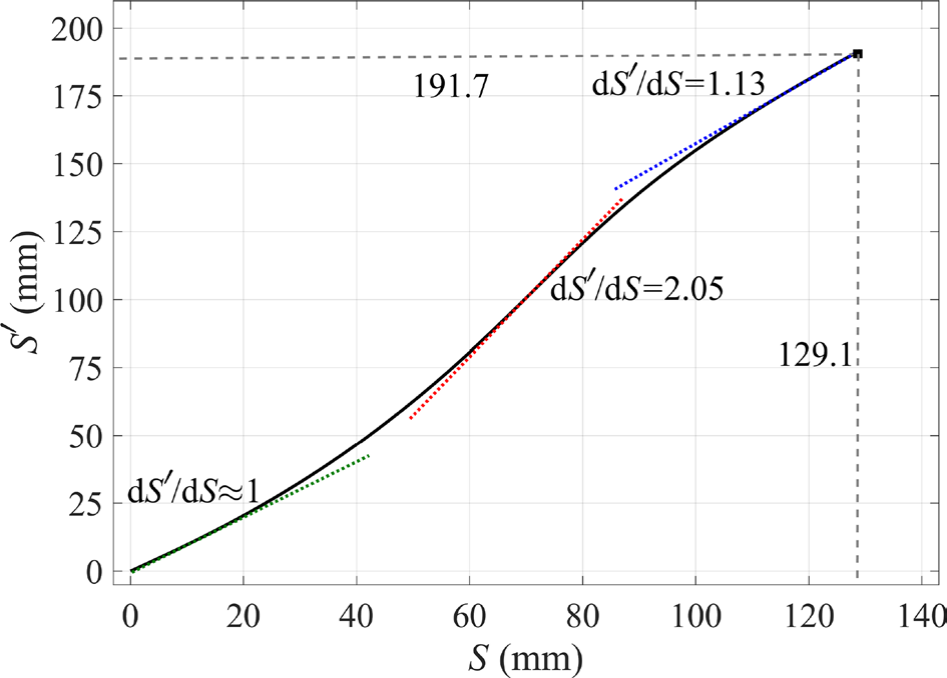
The rescaled optical path function $S'(S)$. The maximum value of $S'$ is 192 mm, which equals the length of the ray $b'c'$ in Fig. 1c. Also the maximum value of *S* is 129 mm that equals the length of the straight ray *bc* in Fig. 1a as required.

Note that the refractive index has changed while the shape of the rays has not. This shows that there can be different index profiles corresponding to the same set of rays. We can now employ this freedom to manipulate the index according to our needs. In particular, we will use it to eliminate the superluminal region in Fig. 1c. Since the index *n* has the lowest values along the curve $b'c'$ (or equivalently, along $a'd'$), we require the index $n'$ to be unity along these curves. Therefore, the rescaled optical path $S'$ along the ray $b'c'$ must equal the length measured along that ray. This, combined with the known original optical path *S* along that ray, fully defines the rescaling $S'(S)$, which is shown in Fig. 2 for our case.

**Figure 3:**
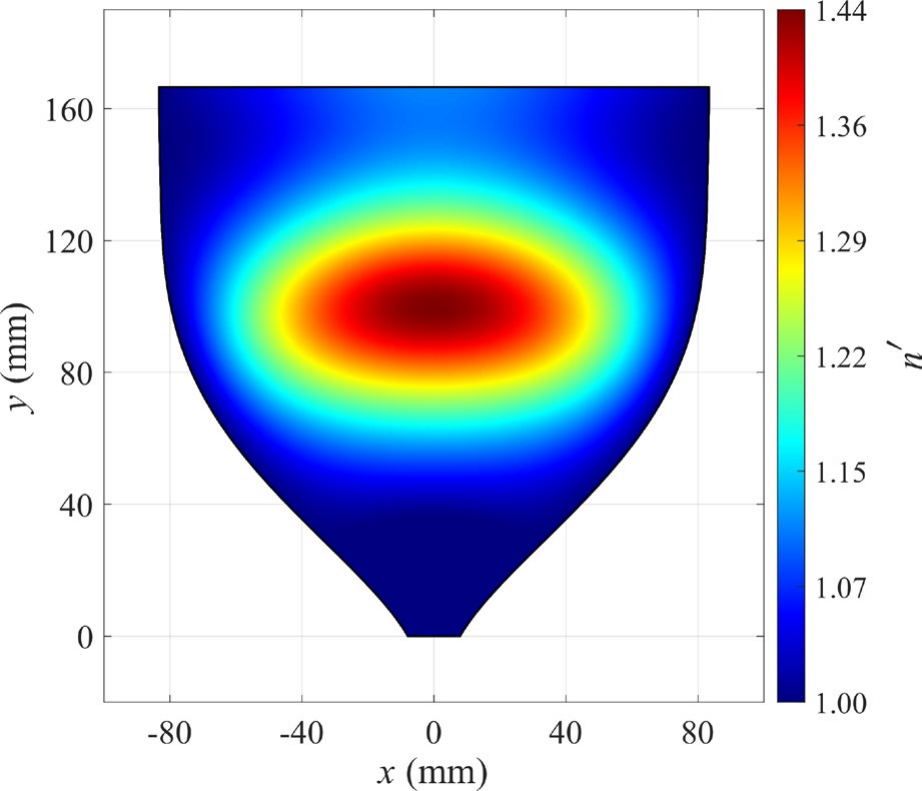
Refractive index of the CTO horn antenna after rescaling the optical path for mitigating the superluminal regions.

When we apply the rescaling function $S'(S)$ to the whole physical space, we get the refractive index $n'(x,y)$ shown in Fig. 3. We see that the superluminal regions have disappeared completely, which was our goal, and the entire refractive index profile ranges from unity to about 1.44.

## Horn lens antenna implementation

**Figure 4:**
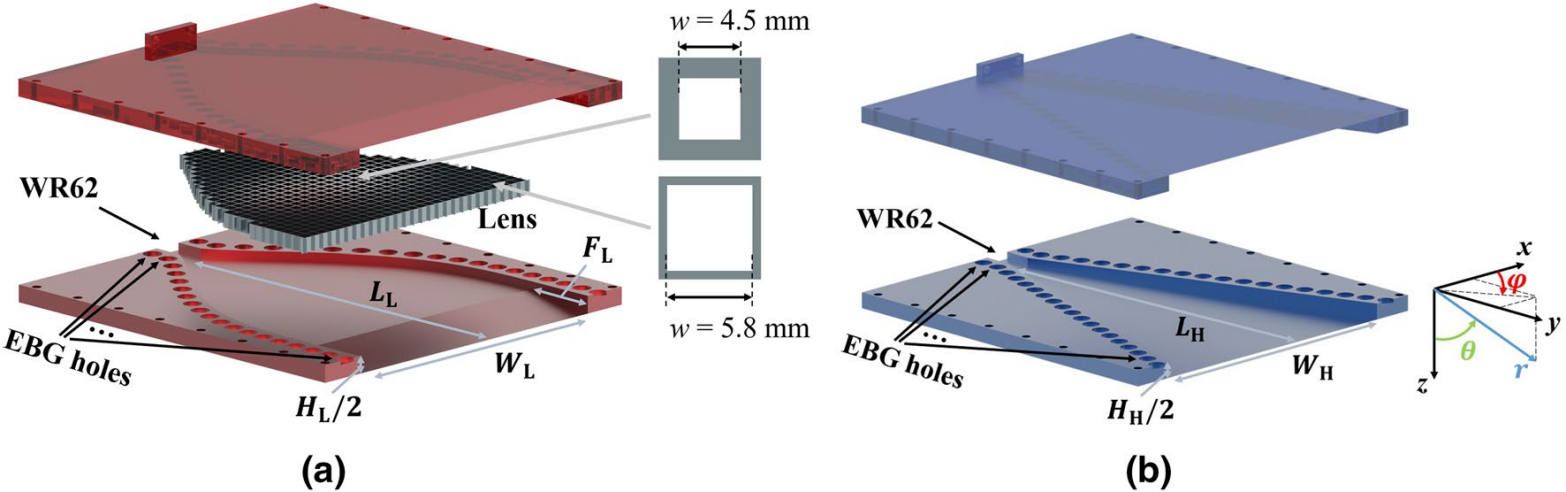
Rendition of (**a**) the H-plane horn lens antenna, and (**b**) the reference H-plane horn antenna.

To validate the proposed method, an H-plane horn lens antenna operating at 15 GHz is designed and manufactured. It is terminated with an E-plane flare to suppress reflections at the aperture of the antenna occurring due to the aperture mismatch. In addition, an H-plane horn antenna is designed and manufactured and serves as a reference. The reference antenna has a slight linear E-plane taper (from the feeding to the aperture) so that the aperture is the same as for the horn lens antenna. The two antennas are illustrated in Fig. 4.

**Figure 5:**
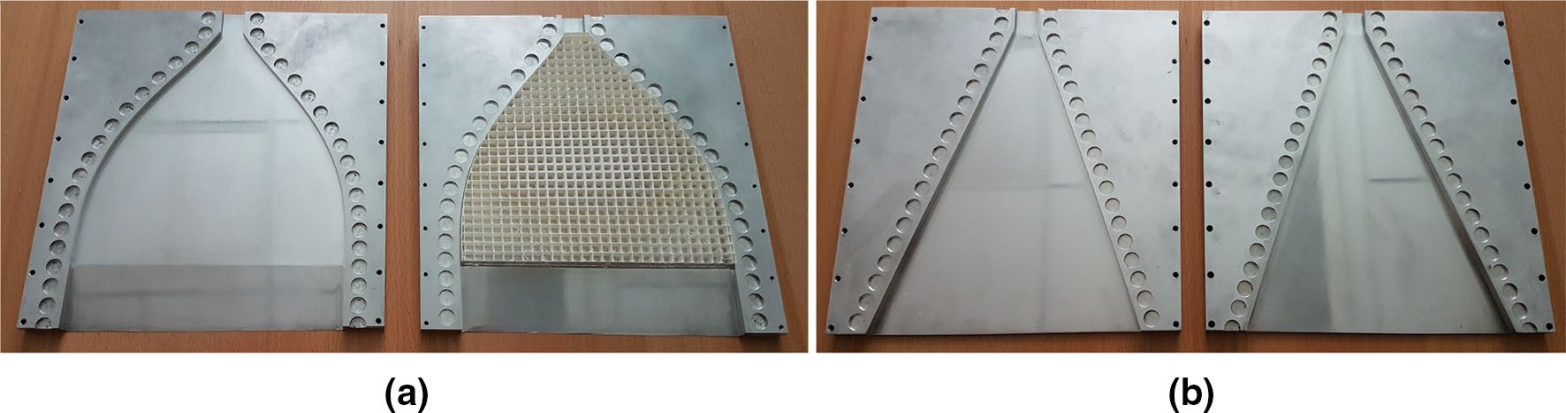
Photograph of (**a**) the H-plane horn lens prototype, and (**b**) the reference H-plane horn prototype.

The two metallic structures are manufactured in two parts each, and the two parts are joined together with screws. A glide-symmetric holey EBG structure is placed along the sidewalls of the structures to avoid leakage^50,51^. The two antennas are fed by a standard WR62 (15.79 mm width and 7.9 mm height) rectangular waveguide. The manufactured prototypes are presented in Fig. 5.

**Figure 6:**
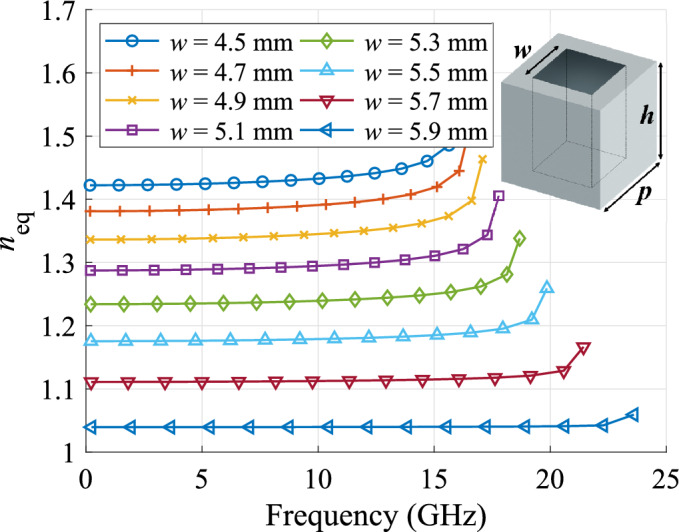
Equivalent refractive index of the perforated dielectric slab ($\varepsilon _r = 3$) for various hole sizes ($p = 6$ mm and $h = 2$ mm).

The phase-correcting lens (highlighted in grey in Fig. 4a) consists of a dielectric slab with periodically spaced square holes. The side of the holes is varied throughout the slab to create the required gradient index profile (Fig. 3). Each hole experiences a quasi-periodic environment. Therefore, the effect of the hole size on the equivalent refractive index can be studied with the *eigenmode solver* of *CST* with periodic boundary conditions. The equivalent refractive index was calculated as the relation between the propagation constant in the periodic structure and the propagation constant in free space^52,53^. The equivalent refractive index versus the frequency is presented in Fig. 6 for various hole size values. Since the maximum required refractive index in the lens is 1.44, a permittivity of 3 is chosen for the dielectric slab. It should be noted that, since a desktop 3D printer is used for manufacturing, the lowest reachable equivalent refractive index is above unity^54^. This results in slightly increased reflections at the borders of the lens.

**Figure 7:**
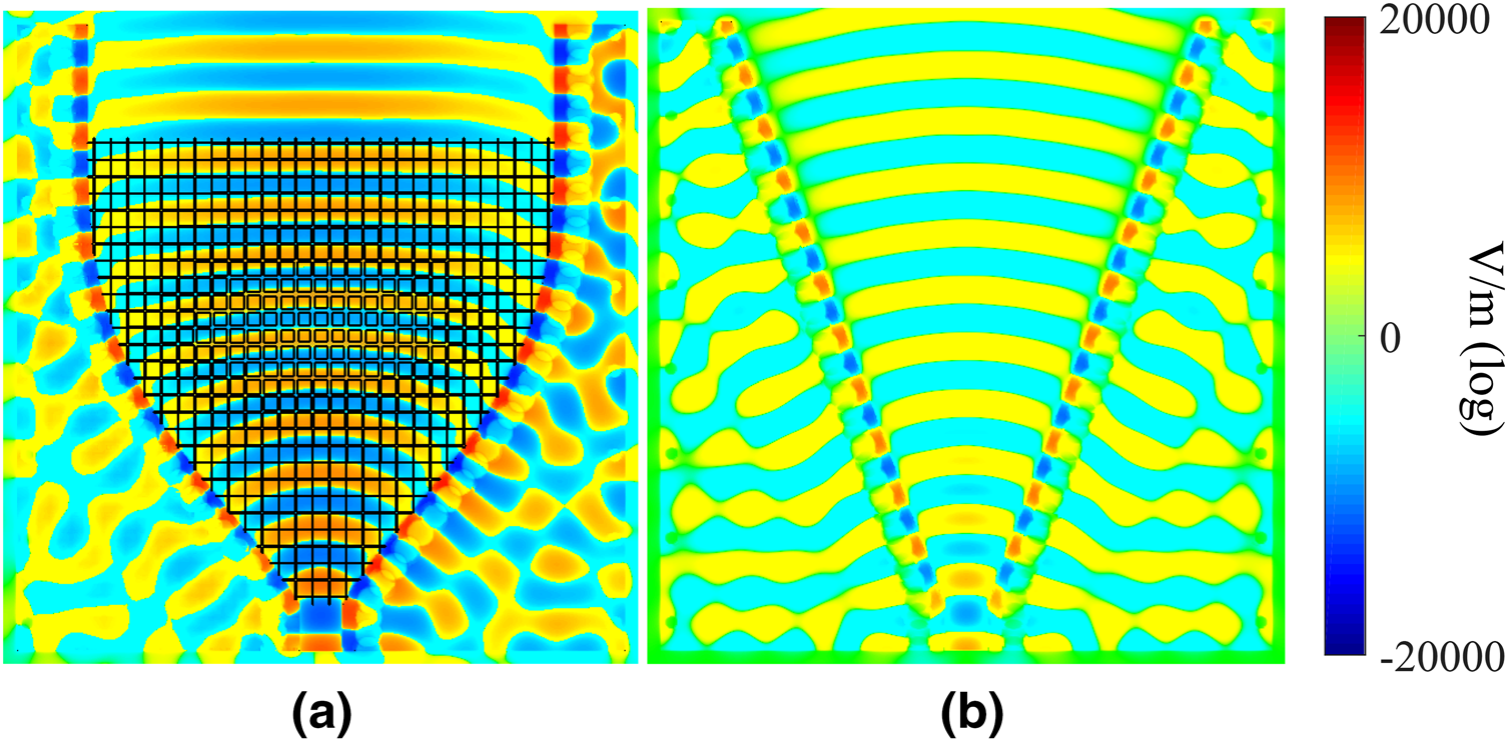
Simulated amplitude distribution of ${E_z}$ for the two H-plane antennas: (**a**) horn lens, and (**b**) reference horn. The dimensions are $L_{\mathrm {L}}=L_{\mathrm {H}}=207$ mm, $W_{\mathrm {L}}=W_{\mathrm {H}}=167$ mm, $H_{\mathrm {L}}=H_{\mathrm {H}}=20$ mm, and $F_{\mathrm {L}}=40$ mm.

**Figure 8:**
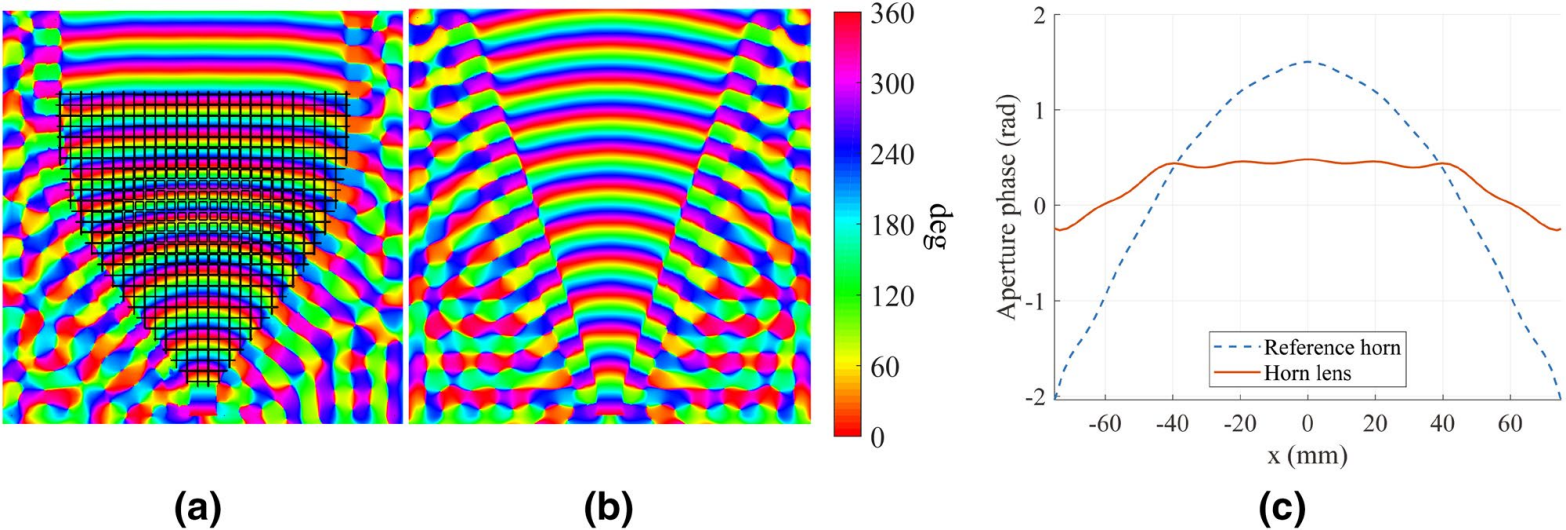
Simulated phase distribution of ${E_z}$ for the two H-plane antennas: (**a**) horn lens, and (**b**) reference horn. (**c**) Aperture phase comparison between the H-plane horn lens (solid red line) and reference horn (dashed blue line). The dimensions are $L_{\mathrm {L}} = L_{\mathrm {H}} = 207$ mm, $W_{\mathrm {L}} = W_{\mathrm {H}} = 167$ mm, $H_{\mathrm {L}} = H_{\mathrm {H}} = 20$ mm, and $F_{\mathrm {L}} = 40$ mm.

The two structures in Fig. 4 are simulated using the *time domain solver* of *CST*, and the E-field amplitude and phase are shown in Figs. 7 and 8. Boundary conditions were assumed to be ‘open add space’ in *CST*, which means radiating boundary conditions. The structure was excited with a ‘waveguide port’ including only one mode, the $\mathrm{{T}}{\mathrm{{E}}_{10}}$, which is the only one above cut-off at this specific frequency. Furthermore, the simulated phase distribution of the electric field at the border of the aperture of both antennas is presented in Fig. 8. It is seen that the lens flattens the phase front at the aperture.

**Figure 9:**
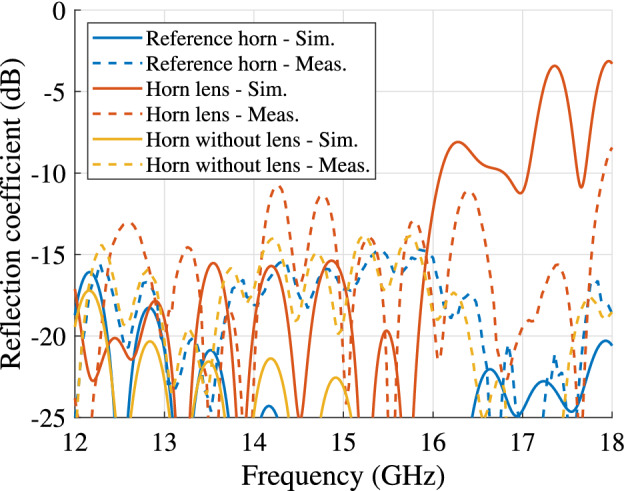
Simulated and measured reflection coefficients for the horn lens antenna, profiled horn antenna without lens, and the reference antenna. The dimensions are $L_{\mathrm {L}} = L_{\mathrm {H}} = 207$ mm, $W_{\mathrm {L}} = W_{\mathrm {H}} = 167$ mm, $H_{\mathrm {L}} = H_{\mathrm {H}} = 20$ mm, and $F_{\mathrm {L}} = 40\hbox { mm}$.

The simulated and measured reflection coefficients are depicted in Fig. 9. Low reflections ($<-10$ dB) are observed in a wide bandwidth for both antennas. The differences in the measured reflection coefficient are due to the tolerances in the lens manufacturing, which was done in-house using a standard desktop 3D printer. In terms of bandwidth, this device is limited by the implementation aspects. More specifically, the lowest frequency of operation is limited by the cut-off frequency of the $\mathrm{{T}}{\mathrm{{E}}_{10}}$ mode in the ${\mathrm{{K}}_{\mathrm{{u}}}}$ band. The standard establishes the minimum frequency in the ${\mathrm{{K}}_{\mathrm{{u}}}}$ band at 12 GHz. The highest frequency is constrained by the operation of the dielectric unit cell (illustrated in Fig. 6) and the quality of the coax-to-waveguide transition (illustrated in Fig. 9). Both aspects are limited to approximately 16 GHz.

**Figure 10:**
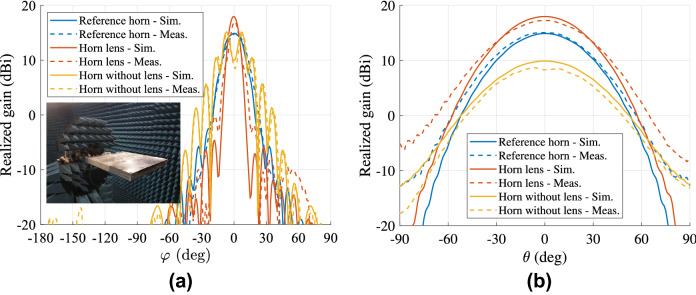
Simulated and measured far-field of (**a**) H-plane ($\theta = 90^\circ$), and (**b**) E-plane ($\varphi = 90^\circ$) at 15 GHz for the horn lens antenna, profiled horn antenna without lens, and the reference antenna. The dimensions are $L_{\mathrm {L}} = L_{\mathrm {H}} = 207$ mm, $W_{\mathrm {L}} = W_{\mathrm {H}} = 167$ mm, $H_{\mathrm {L}} = H_{\mathrm {H}} = 20$ mm, and $F_{\mathrm {L}} = 40$ mm.

**Figure 11:**
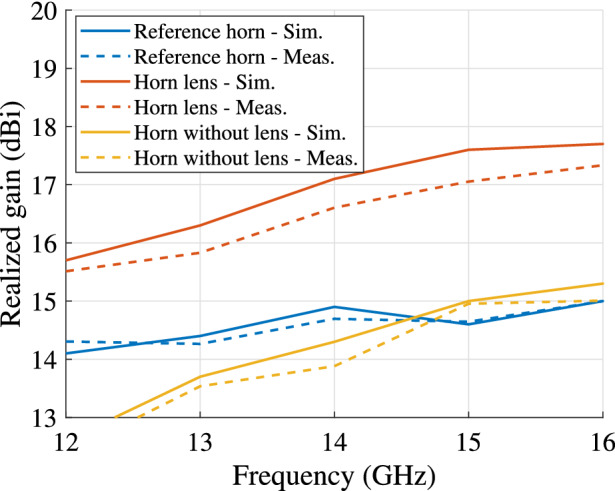
Simulated and measured realized gain for the horn lens antenna, profiled horn antenna without lens, and the reference horn antenna. The dimensions are $L_{\mathrm {L}} = L_{\mathrm {H}} = 207$ mm, $W_{\mathrm {L}} = W_{\mathrm {H}} = 167$ mm, $H_{\mathrm {L}} = H_{\mathrm {H}} = 20$ mm, and $F_{\mathrm {L}} = 40$ mm.

The simulated and measured far-field (two cuts) at 15 GHz are presented in Fig. 10. The gain versus the frequency for the two antennas is illustrated in Fig. 11. The horn lens antenna has a higher peak gain than the reference horn antenna over a wide bandwidth (1.5-2.4 dB higher measured gain in the frequency range 12-16 GHz). This gain enhancement corresponds to an increase of the effective aperture area by a factor of 1.3-1.7. A comparison is also made between the horn lens and the profiled horn without the lens embedded. The comparison is presented in Figs. 9, 10 and 11.

## Conclusion

In this work, we demonstrated a design methodology for a GRIN lens that corrects the phase error in the aperture of a conventional horn antenna. Our design considerations avoid the singular index values and employ an optical path rescaling to mitigate the need for a sub-unity refractive index. Here, we focused on enhancing the directivity of a horn antenna by designing a conformal lens. However, our methodology can be used to design various GRIN elements, including beam compressors and expanders, waveguide couplers, and plasmonic cloaks.
